# Dynamic regulation and targeted interventions of macrophages in ischemia–reperfusion injury

**DOI:** 10.1016/j.jare.2025.05.006

**Published:** 2025-05-10

**Authors:** Ping Lu, Ruotong Shen, Jingjing Yang, Longlong Wu, Rong Wang

**Affiliations:** The Gastroenterology Department of Shanxi Provincial People’s Hospital, Shanxi Medical University, Taiyuan 030012, China

**Keywords:** Macrophage, Ischemia-reperfusion injury, Programmed cell death, Immunometabolism, Therapeutic application

## Abstract

Ischemia-Reperfusion Injury (IRI) is a complex pathophysiological process characterized by oxidative stress and inflammatory responses during tissue reperfusion, leading to severe organ dysfunction. Macrophages, as key immune cells, play a pivotal role in the pathogenesis of IRI, exhibiting dynamic functions that influence both tissue damage and repair. Despite extensive research, the precise mechanisms underlying macrophage-mediated IRI remain incompletely understood, necessitating a comprehensive review to explore their multifaceted roles and potential therapeutic targets.

Aim of Review: This review aims to elucidate the diverse roles of macrophages in IRI, focusing on their involvement in programmed cell death mechanisms, communication with other immune cells, and regulatory effects on key organs affected by IRI. The review also explores potential therapeutic strategies targeting macrophages to mitigate IRI-induced injury.

Key Scientific Concepts of Review: This article reviews the multifaceted roles of macrophages in IRI and explores various modes of macrophage programmed cell death induced by IRI, including gasdermin D-mediated pyroptosis, lipid peroxidation-associated ferroptosis, PARP-1-mediated PAR-dependent cell death, PANoptosis regulated by the PANoptosome, and the formation of macrophage extracellular traps (METs) induced by both reactive oxygen species-dependent and −independent pathways. Additionally, it discusses intercellular communication between macrophages and other immune cells in IRI, focusing on the bidirectional regulatory effects between macrophages and neutrophils, as well as their synergistic role in resolving inflammation. Moreover, the regulatory mechanisms of macrophages in IRI affecting key organs, such as the brain, lung, heart, kidneys and liver, have been systematically summarized. Finally, innovative therapeutic strategies targeting macrophages, including precise approaches such as regulating cell polarization, inhibiting excessive METs formation, and utilizing nano-drug delivery systems, are thoroughly analyzed. This review provides a significant theoretical foundation for clinical translational research on IRI.

## Highlights

• Macrophages are crucial in IRI progression and recovery.
• Key mechanisms of macrophage death in IRI.
• Macrophage-immune cell interactions shape inflammation and tissue repair in IRI.
• Therapeutic strategies target macrophage polarization and extracellular traps.
• Organ-specific macrophage roles in IRI affect the heart, kidneys, liver, and brain.
• Nano-drug delivery systems are advancing therapies targeting macrophages in IRI.

## Graphical abstract

**Figure:**
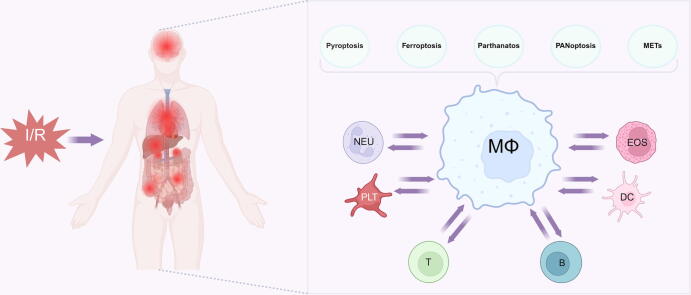

## Introduction

Ischemia-Reperfusion Injury (IRI) refers to the functional and structural alterations that occur when blood flow is restored following a period of ischemia. IRI is not only a key factor in the pathological progression of many diseases but also contributes to delayed graft recovery. Although the role of IRI has been extensively studied in various organs, the precise mechanisms and pathways involved remain poorly understood and are highly contentious. Beyond ischemia, reperfusion itself can exacerbate tissue and organ damage, particularly through inflammatory processes. Under normal conditions, macrophages protect the body from infection and regulate tissue inflammation. During ischemia, macrophages are activated by diverse signals and initiate an inflammatory response by releasing oxygen species (ROS), pro-inflammatory cytokines, and chemokines. Upon reperfusion, macrophages accumulate at the injury site, where they exert a dual function. On the one hand, they exacerbate inflammation and oxidative stress by producing cytokines, thereby aggravating tissue damage and dysfunction [[1], [2]]. On the other hand, they facilitate tissue repair and regeneration by clearing cellular debris, promoting angiogenesis, and releasing growth factors that support tissue healing [[3], [4]]. Therefore, gaining a comprehensive understanding of the molecular mechanisms underlying macrophage-mediated tissue damage and repair during IRI, as well as developing targeted therapeutic strategies, is essential for the clinical prevention and treatment of IRI.

## Macrophage

### Origin

Macrophages are mature white blood cells responsible for phagocytosis, a process that eliminates pathogens. They play a pivotal role in the body’s innate immune response, acting as the first line of defense against pathogen invasion. Tissue-resident macrophages can originate from hematopoietic stem cells in the bone marrow or from progenitor cells during embryonic development in the fetal liver, yolk sac, or regions adjacent to the dorsal aorta [5]. Furthermore, some macrophages may arise through extramedullary hematopoiesis, as observed in the spleen. Due to their widespread distribution and diverse functional roles across tissues and organs, macrophages display a variety of morphologies and phenotypes.

### Macrophage-Associated programmed cell death in IRI

In response to external stimuli and challenges, macrophages induce programmed cell death, or apoptosis, through the activation of specific signal transduction pathways. Apoptosis is a highly regulated form of cell death that modulates the intensity and duration of the inflammatory response by controlling the release of inflammatory mediators. In addition to apoptosis, macrophages also undergo other forms of programmed cell death, including pyroptosis, ferroptosis, PARP-1-dependent cell death, and necroptosis. These cell death mechanisms are also implicated in organ IRI.

#### Pyroptosis

Pyroptosis is a unique form of inflammatory cell death with diverse molecular mechanisms. In the classical pathway, pathogens induce macrophages to cleave GSDMD by activating Caspase-1. Recent studies have shown that Caspase-8 can also cleave gasdermin D (GSDMD), a process induced by the inhibition of cell survival signals, particularly through targeting the TAK1 kinase [6]. Once cleaved, the amino-terminal domain of GSDMD becomes free and rapidly integrates into the cell membrane, disrupting its integrity and causing an imbalance in osmotic pressure between the intracellular and extracellular environments. This membrane rupture leads to the release of a large quantity of inflammatory cytokines, including members of the IL-1 family and damage-associated molecular patterns (DAMPs), which significantly exacerbate the inflammatory cascade triggered by IRI in organs. In addition to GSDMD, Caspase-3 can also induce the cleavage of gasdermin E (GSDME) [7], and both GSDMD and GSDME target the mitochondrial membrane, stimulating the release of reactive ROS. A recent study by Li et al. [8] demonstrated that Maresin 1, a bioactive lipid derived from docosahexaenoic acid, effectively reduces pyroptosis in liver macrophages, thereby alleviating liver IRI by stabilizing mitochondrial membrane potential and inhibiting cytochrome *C* release.

**Fig. 1:**
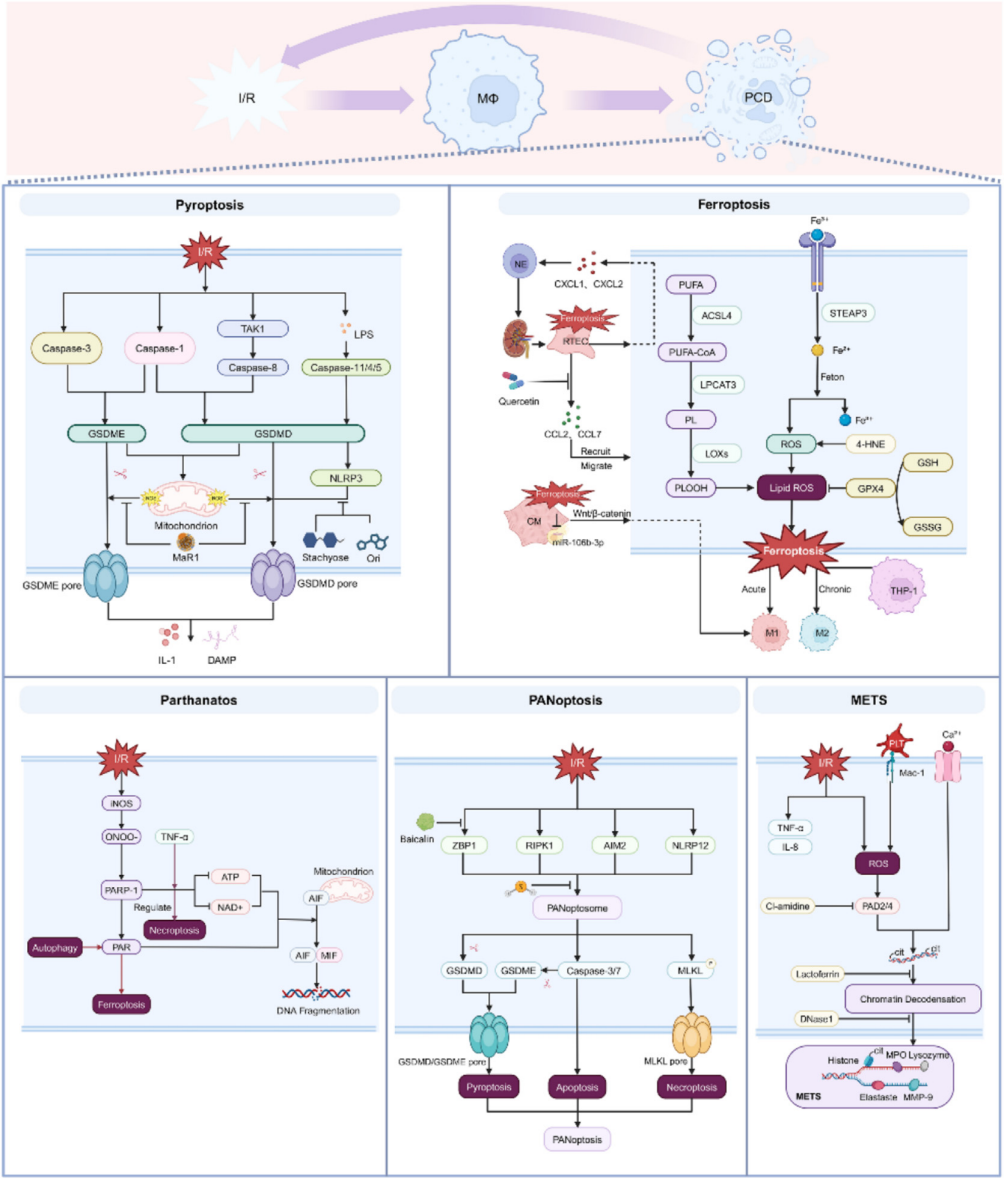
Macrophage-mediated programmed cell death in IRI. The primary macrophage-associated programmed cell death mechanisms in ischemia–reperfusion injury include pyroptosis, ferroptosis, parthanatos, PANoptosis, and METs. Each mechanism is characterized by its distinct molecular interactions and downstream effects. This illustration emphasizes their roles in inflammation and tissue damage, highlighting potential therapeutic strategies to alleviate IRI.

The complexity of the pyroptosis mechanism is also reflected in the atypical pathway mediated by Caspase-11/4/5. This non-classical inflammasome is activated by cytosolic lipopolysaccharide (LPS); however, when sensing cytosolic LPS through this pathway, IL-1β production relies on the synergistic action of NOD-like receptor protein 3 (NLRP3) [9]. NLRP3-mediated macrophage pyroptosis is closely associated with hepatic IRI. Oridonin, a covalent inhibitor of NLRP3, exerts a significant anti-inflammatory effect by inhibiting the interaction between the M2 subtype of pyruvate kinase and NLRP3 in hypoxic/reoxygenated macrophages [10], thereby reducing liver macrophage pyroptosis and alleviating liver IRI. Recently, Lin et al. found that quercetin inhibited the pyrodeath of Caspase-8/ ASC-dependent macrophages and alleviated hepatic IRI [11]. In a study targeting macrophages therapeutically, Zhang et al. found that a natural stachyose inhibits the pyroptosis pathway in macrophages by downregulating the expression of NLRP3, GSDMD-N, and Caspase-1. This results in reduced levels of the pro-inflammatory cytokines IL-1β and IL-18, ultimately leading to significant improvements in cardiac function in IRI mice [12]. However, when exposed to complex physiological and pathological stimuli, cells do not passively choose a single mode of death; rather, they exhibit diversity and plasticity (Fig. 1). This biological property allows cells to flexibly adjust their death strategies in response to different environmental signals and varying degrees of damage.

#### Ferroptosis

Ferroptosis is a form of programmed cell death characterized by lipid peroxidation. During ferroptosis, ferrous ions produce large amounts of reactive ROS through the Fenton reaction, while glutathione peroxidase 4 (GPX4) and glutathione (GSH) finely regulate ROS levels [13]. When GPX4 or GSH is depleted, lipid peroxidation products, such as 4-hydroxy-2-nonenal, accumulate [14], leading to a rapid increase in intracellular ROS levels and triggering a widespread lipid peroxidation cascade. Additionally, the overactivation of acyl-CoA synthetase long-chain family member 4 and lipoxygenases can convert polyunsaturated fatty acids into lipid peroxides [15], thereby accelerating cell membrane damage. Ferroptosis significantly influences the polarization and immune function of macrophages through a delicate metabolic reprogramming mechanism. It induces iron overload in macrophages, promotes polarization toward the M1 phenotype, enhances the secretion of inflammatory factors, and inhibits tissue repair and immunomodulatory capacity [16]. However, under conditions of chronic iron overload, THP-1 monocyte-derived macrophages can be converted to the M2 phenotype [17], a process that is accompanied by significant downregulation of M1 macrophage marker molecules.

The complexity of iron metabolism regulation extends beyond the transformation of the macrophage phenotype; it also involves the extensive modulation of the immune microenvironment through the activation of molecular inflammatory pathways in cells undergoing ferroptosis (Fig. 1). Recently, Wang et al. demonstrated that quercetin reduces CCL2 and CCL7 levels by inhibiting ferroptosis in proximal tubular epithelial cells, thereby hindering the recruitment and chemotaxis of macrophages and alleviating renal IRI [18]. Additionally, iron-induced cell death can trigger macrophages to release CXCL1 and CXCL2, which attract neutrophils to the kidney. Inhibition of iron death can prevent macrophage and neutrophil infiltration in the kidney and block renal IRI [19]. Similar to the kidney, in a myocardial infarction model, ferroptosis reduces the content of exosomal miR-106b-3p secreted by cardiomyocytes, thereby activating the Wnt/β-catenin signaling pathway and ultimately polarizing macrophages toward an M1-like phenotype, further amplifying the inflammatory response [20]. These findings suggest that ferroptosis plays a critical role in maintaining immune homeostasis, modulating autoimmune responses, and promoting tissue repair.

#### Parthanatos

Parthanatos is a form of cell death mediated by poly (ADP-ribose) (PAR). There is a complex interaction between Parthanatos and macrophages. Zingarelli et al. [21] demonstrated that exposure of mouse macrophages to high concentrations of LPS triggers rapid superoxide production and a gradual upregulation of inducible nitric oxide synthase, creating favorable conditions for the formation of peroxynitrite. This, in turn, activates poly (ADP-ribosome) polymerase 1 (PARP-1), leading to the depletion of NAD + and ATP. However, this process is not merely a passive disorder of energy metabolism; it also involves the abnormal activation of apoptosis-inducing factor (AIF). Recent studies have shown that PARP-1-mediated NAD + depletion induces mitochondrial membrane potential depolarization, promotes the release of AIF from mitochondria to the cytoplasm, and interacts with macrophage migration inhibitory factor (MIF) [22], ultimately resulting in cell death. This cascade is widely observed in various diseases, such as neurodegenerative damage, stroke, and heart failure. Studies have found [23] that baicalin can significantly inhibit the release of cytokines, PARP-1 activation, and the nuclear translocation of MIF in brain IRI rats, thereby reducing ischemic damage to brain tissue. Additionally, the use of PARP-1 inhibitors, Iduna protein [24], and activated protein C [25] can effectively alleviate IRI. PARP-1 inhibitors not only directly protect neurons by blocking the cell death cascade but also reduce the nuclear factor kappa-B (NF-κB)-mediated pro-inflammatory response of microglia, inhibit the release of matrix metalloproteinase, and limit blood–brain barrier (BBB) damage and hemorrhagic transformation [26], effectively controlling secondary damage from the inflammatory process.

There is an inseparable and intrinsic connection between non-apoptotic programmed cell death mechanisms (Fig. 1). TNF-α precisely regulates necroptosis by inducing ATP depletion and activating PARP-1. Autophagy, a key cellular stress response mechanism, also plays a critical role in parthanatos. During autophagy, PARP-1 activity remains relatively stable, while large amounts of PAR are synthesized. This process also promotes ferroptosis [27]. These mechanisms work together to finely regulate macrophages.

#### Panoptosis

PANoptosis, a novel form of programmed cell death, integrates the key features of pyroptosis, apoptosis, and necroptosis, while also exhibiting unique regulatory mechanisms and biological characteristics (Fig. 1) [28]. Its core mechanism involves the formation and activation of the PANoptosome complex, which consists of three types of proteins: Z-DNA binding proteins, which recognize and capture pathogen-associated molecular patterns (PAMPs) and DAMPs; speck-like proteins and Fas-associated proteins, which facilitate protein–protein interactions and signal transduction through their specific domains; and receptor-interacting protein (RIP), which acts as an effector protein to mediate key signal transduction and regulate cell death [29]. The activation of this complex is controlled by upstream molecules such as ZBP1, RIPK1, AIM2, and NLRP12 [30], which detect specific stress signals and trigger downstream effects leading to cell death.

It is important to note that this pathway plays a crucial role in various pathological processes, including IRI in the brain [31], retina [32], and spinal cord. In a study of retinal precursor cells (R28), Yan et al. [33] demonstrated that IRI induced significant morphological and protein changes in retinal neurons by upregulating the expression of caspase-1, caspase-8, and NLRP3, thus confirming the presence of PANoptosis-like cell death in both in vitro and in vivo environments. Although this study primarily focused on local neurons, it provided valuable insights into the mechanisms of cell death in the nervous system. In this context, Xie et al. [34] identified that among many signal transduction pathways, hydrogen sulfide acts as a key regulatory factor that can effectively inhibit neuronal apoptosis in spinal cord IRI rats by modulating the polarization of microglia and macrophages, demonstrating a potential neuroprotective effect. Although targeting PANoptosis represents a promising therapeutic strategy, effective PANoptosis inhibitors remain scarce. Baicalin, an active flavonoid isolated from traditional Chinese herbs, has been shown by You et al. [35] to inhibit PANoptosis in macrophages by blocking the formation of mitochondrial Z-DNA and the assembly of the ZBP1-PANoptosome, thereby reducing the inflammatory response. Further research is needed to elucidate how the components of the PANoptosome interact within a single complex, and to explore in greater depth the upstream and downstream regulatory mechanisms of this pathway.

#### Macrophage extracellular traps

In addition to classical non-apoptotic programmed cell death mechanisms, macrophages possess a unique active defense mechanism. When exposed to pathogens or chemical stimuli, macrophages trigger a series of pro-inflammatory responses, leading to the production of ROS and the release of pro-inflammatory cytokines such as TNF-α and IL-8. This inflammatory microenvironment activates peptidylarginine deiminase (PAD2 or PAD4), which translocates to the nucleus and catalyzes histone guanylation, inducing chromatin depolymerization [36]. In turn, macrophages release a network of histones, elastase, myeloperoxidase, matrix metalloproteinase-9, and lysozyme, collectively known as macrophage extracellular traps (METs) [37]. Beyond pathogens and chemical stimuli, heme-activated platelets can also interact with macrophages via Mac-1 and other intercellular adhesion molecules [38], ultimately triggering METs formation. However, ROS is not a necessary condition for METs formation. Human monocyte-derived macrophages exposed to pathological levels of hypochlorous acid (HOCl) induce METs formation, accompanied by the release of mitochondrial DNA and nuclear DNA [39]. During this process, the concentration of Ca^2+^ increases significantly, suggesting that Ca^2+^ influx may promote METs formation via a ROS-independent pathway. This study also proposed that inflammatory M1 macrophages are more prone to forming METs, while selectively activated M2 macrophages are less susceptible to HOCl-induced damage. Subsequent studies confirmed that the formation of METs in M1 macrophages was significantly increased in response to stimuli such as phorbol myristate acetate, TNF-α, IL-8, and IFN-γ [40]. These findings reveal the functional plasticity of macrophages in different microenvironments and their complex role in inflammation regulation.

Through the formation of a mesh structure, METs can not only effectively capture pathogens and limit their spread but also contribute to pathogen killing [41]. Additionally, METs can work synergistically with other innate immune defense mechanisms, such as complement proteins, to enhance infection clearance. However, METs can also act as a scaffold for pathogen aggregation, which may, to some extent, promote bacterial survival in host tissues [42]. This dual effect suggests that METs play a complex role in both host defense and pathogen control. While most studies focus on the relationship between METs and infection, recent research has revealed that METs are also involved in cellular functions, including lipid and iron metabolism, and are closely linked to sterile inflammation and IRI [43].Okubo et al. [38] demonstrated that in rhabdomyolysis, heme released from necrotic muscle cells induces METs formation by activating platelets, which ultimately leads to acute kidney injury (AKI). Liver macrophages account for approximately 15 % of all liver cells [44] making liver METs highly likely to cause severe inflammatory damage. Wu et al. found that under hypoxic conditions, the phagocytic function of macrophages is impaired, while METs formation is increased. Inhibiting METs formation reduces ferroptosis in hepatocytes, thereby improving hepatocyte survival after hypoxia [45].As the significant role of METs in various diseases is increasingly recognized, targeting METs release has emerged as a potential therapeutic strategy. For example, the pan-PAD inhibitor Cl-amidine has been shown to improve hepatocyte survival after hepatic IRI by inhibiting METs release [45]. Lactoferrin inhibits METs formation by preventing macrophage membrane rupture and promoting chromatin condensation [46]. However, similar to Cl-amidine, this strategy may lack specificity, as lactoferrin also inhibits the release of neutrophil extracellular traps (NETs). Additionally, DNase1 has been shown to successfully degrade METs both in vitro and in vivo [47]. These findings offer new insights into the treatment of IRI and hold significant clinical translational potential.

## Macrophage interactions with other cells

Macrophages communicate with each other through various mechanisms, including direct cell-to-cell contact, the secretion of signaling molecules such as cytokines and chemokines, and the exchange of extracellular vesicles. This intercellular communication enables macrophages to coordinate their responses to pathogens, tissue damage, and other stimuli, thereby ensuring an effective immune response and promoting tissue repair. Furthermore, macrophages can interact with other cells to regulate their own activation state, which allows them to amplify or modulate inflammatory or anti-inflammatory signals. This regulation also influences the behavior of other immune cells within the surrounding microenvironment. Such a complex communication network ensures that macrophages can generate appropriately coordinated responses, helping to maintain tissue homeostasis and protect the host from infection and disease.

### Neutrophils

Neutrophils are among the first effector cells recruited during the inflammatory response and play a crucial role in the pathological process of IRI. Under normal conditions, tissue-resident macrophages perform an essential immune surveillance function by quickly identifying and isolating necrotic cells. This helps block chemokine-mediated feedforward signaling cascades, thereby preventing excessive neutrophil recruitment and limiting inflammatory damage [48]. Additionally, this “immune invisibility” mechanism helps maintain tissue homeostasis during everyday damage, such as mechanical stress. However, under hypoxic stress, tissue-resident macrophages become activated and release a variety of DAMPs, such as HMGB1, S100 proteins, heat shock proteins, and circulating DNA/RNA. Among these, HMGB1 plays a pivotal role as a DAMP molecule, triggering the recruitment of MyD88 via TLR4, which further induces the production of pro-inflammatory cytokines like TNF-α and IL-6. This cascade enhances the expression of adhesion molecules on vascular endothelial cells, facilitating neutrophil migration to the site of injury [49,50].In addition to this classical inflammatory pathway, recent studies have identified C-type lectin receptors as key players in DAMPs recognition and regulation of inflammatory responses. Notably, the deletion of CLEC-1 produces a dual effect: on the one hand, it reduces CCL2 expression, limiting the accumulation of monocyte-derived macrophages post-injury and impairing liver regeneration; on the other hand, CLEC-1 deficiency results in excessive TNF-α and IL-1β production by Kupffer cells, promoting early neutrophil infiltration and exacerbating collateral tissue damage [51].Similarly, nerve injury-induced protein 1 (Ninj1) plays an important role in modulating immune responses during various inflammatory processes [52]. In the context of liver IRI, Ninj1 mediates the pro-inflammatory response of Kupffer cells via the DUSP1 signaling pathway and influences neutrophil infiltration by regulating the expression of chemokine CXCL1. This finding offers new insights into the regulatory network of the tissue inflammatory microenvironment.

**Fig. 2:**
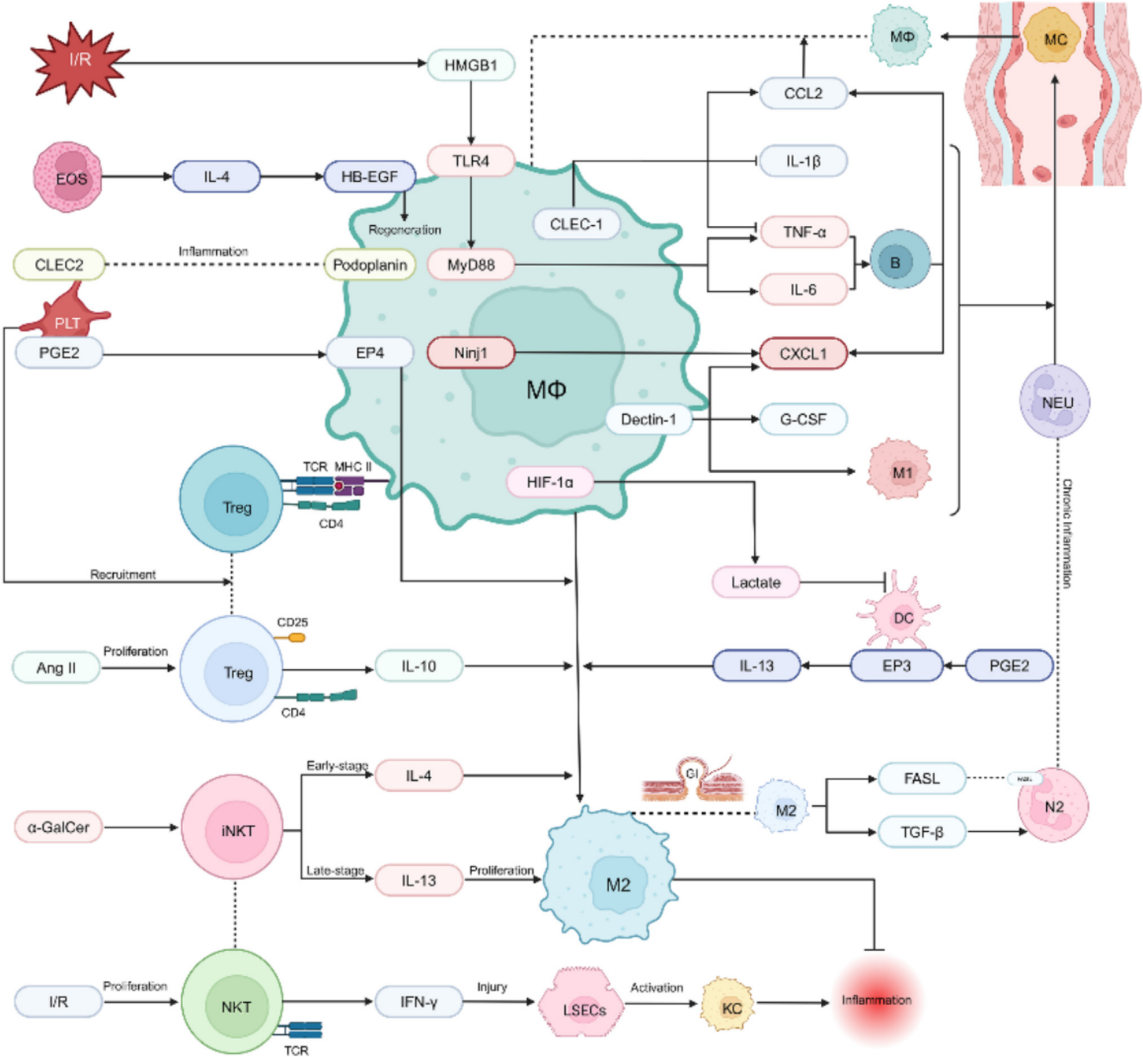
Macrophage interactions with other cells in IRI. This diagram highlights the critical interactions between macrophages and other cells, such as neutrophils, Tregs, NKT cells, and platelets, within the context of IRI. It illustrates how macrophages integrate signals from these cells, influencing their polarization and activity, while playing a pivotal role in regulating the inflammatory response and facilitating tissue repair through pathways involving key molecules like IL-4, HMGB1, and PGE2.

Soon after neutrophils extravasate into inflamed tissues, blood monocytes are recruited in large numbers and differentiate into macrophages and dendritic cells (DCs). Local tissue signals significantly influence the functional remodeling of macrophages (Fig. 2) [53]. Dectin-1, a pattern recognition receptor predominantly expressed on macrophages, is upregulated during the early stages of myocardial IRI. Studies have shown that Dectin-1 induces macrophage polarization toward the M1 phenotype, resulting in the release of pro-inflammatory cytokines, including TNF-α, IL-1β, and IL-23, which promote neutrophil infiltration and exacerbate myocardial injury [54]. Additionally, Dectin-1 deficiency significantly reduces macrophage expression of CXCL1 and G-CSF, while neutrophils infiltrating the heart exhibit high expression of CXCR2 and G-CSFR. These findings further support the hypothesis that the Dectin-1-dependent CXCL1/G-CSF pathway plays a crucial role in myocardial IRI by modulating macrophage and neutrophil interactions. Moreover, neutrophils have been shown to aid cardiac healing after myocardial IRI by promoting macrophage polarization toward a repair phenotype through the release of lipoproteins associated with neutrophil gelatinase [55]. While neutrophils are traditionally thought to exacerbate tissue damage by releasing proteases and oxidants, recent studies indicate that they also produce important anti-inflammatory and pro-resolving lipid mediators that promote inflammation resolution [56]. During chronic inflammation, neutrophils can adopt an immunosuppressive “N2” phenotype, characterized by reduced Fas ligand expression. Furthermore, TGF-β blockade leads to a decrease in N2 neutrophils, suggesting that TGF-β plays a key role in inducing neutrophil immunosuppression [57]. Notably, M2 macrophages in the intestine are the primary source of TGF-β and Fas ligand. However, whether changes in the neutrophil phenotype also occur in other immune microenvironments within host tissues, and whether this process is dependent on tissue-resident macrophages, remains to be further investigated.

### T lymphocytes

Regulatory T cells (Tregs) are a subset of T lymphocytes characterized by the expression of Foxp3, CD4, and CD25, and are crucial for maintaining immune tolerance. In a myocardial IRI model induced by angiotensin II infusion, the infiltration of CD4 + CD25 + Treg cells was found to increase. Subsequent studies revealed that Tregs contribute to macrophage polarization toward the M2 phenotype following myocardial infarction by producing the anti-inflammatory cytokine IL-10, thereby reducing cardiac hypertrophy, inflammation, and fibrosis [58]. In contrast, Treg cell deficiency leads to a lack of interferon expression in the damaged myocardium, which negatively affects other immune cell populations, including macrophages and neutrophils, ultimately impairing neovascularization and collagen deposition. Additionally, studies have shown that ischemic injury in MHC class II-deficient mice, as well as in mice expressing a single transgenic T cell receptor, results in impaired wound healing [59]. These findings suggest that self-antigens are presented to CD4 + Tregs via MHC class II molecules on cells such as macrophages and dendritic cells, triggering an immunosuppressive response in the heart. Tregs, therefore, play a pivotal immunoregulatory role in cardiac inflammation and repair, promoting cardiac regeneration and remodeling by influencing macrophage polarization and neutrophil function (Fig. 2).

Natural killer T (NKT) cells are a unique subset of T cells that possess both T cell receptors and NK cell receptors on their surface. Activated NKT cells play a crucial role in liver damage, particularly after liver transplantation or resection, during which the number of liver NKT cells increases. This increase in NKT cell numbers is influenced by various factors, including activation-induced cell death, loss of specific NKT cell surface markers [60], apoptosis, and sympathetic nerve activation. On one hand, NKT cells can cause liver cell damage through direct cytotoxicity or the secretion of IFN-γ, further activating Kupffer cells and exacerbating the liver’s inflammatory response [61]. In NKT cell knockout models, liver IRI is significantly reduced, suggesting that NKT cell contributes to liver injury. On the other hand, activated NKT cells (iNKT cells) are involved in the fine regulation of tissue repair through functional interactions with macrophages. For instance, iNKT cells can induce macrophage activation and phenotypic polarization, promoting liver repair [62]. In the early stages of injury, iNKT cells secrete the anti-inflammatory cytokine IL-4, inducing macrophages to adopt a reparative phenotype. In the later stages, proliferating iNKT cells predominantly secrete IL-13, which promotes the expansion of reparative macrophages, thus facilitating liver repair and the resolution of inflammation [63,64]. These findings underscore the crucial role of T cell-macrophage interactions in both cardiac and hepatic IRI. This finely tuned immune regulatory mechanism is essential for maintaining the dynamic balance between tissue damage and repair.

### Platelets

Platelets play a crucial role as immune regulators within the circulatory system. They not only release a variety of immune mediators to modulate inflammatory responses but also participate in tissue repair and remodeling through interactions with immune cells (Fig. 2). Specifically, prostaglandin E2 derived from platelets can induce macrophages to adopt an anti-inflammatory phenotype by activating the EP4 receptor on macrophages [65]. Recently, Kapur et al. [66] found that platelets can recruit regulatory T cells, leading to increased levels of IL-10 and TGF-β, which promote macrophage polarization toward an anti-inflammatory phenotype and reduce tissue inflammation. In these immunomodulatory processes, CLEC2 (C-type lectin-like receptor 2), a key receptor on the platelet surface, and its ligand podoplanin, which is upregulated on macrophages during inflammation, play pivotal roles. Studies have demonstrated that the CLEC2-podoplanin signaling axis is essential in brain IRI models [67], and clinical investigations have found that elevated plasma CLEC2 levels correlate with poor outcomes in patients with ischemic stroke. Additionally, in liver IRI, blocking the interaction between macrophage podoplanin and platelet CLEC2 promotes the recovery of hepatocyte function [68]. These findings highlight CLEC2 as a promising therapeutic target that may not only improve IRI outcomes but also minimize the risk of bleeding.

In recent years, as research on the role of macrophage phenotypes in inflammation regulation has advanced, researchers have found that the peak of monocyte/macrophage recruitment typically occurs after the increase in vascular permeability, making it more difficult to reprogram macrophages through systemic administration. Additionally, current nanomaterials face challenges in effectively releasing their contents to specific intracellular locations, limiting the potential for macrophage reprogramming. Inspired by the accumulation of platelet and monocyte aggregates in the circulation following IRI in patients, Tan et al. constructed a platelet-like fusion liposome (PLP) [69]. Mesoporous silica nanospheres, encapsulated in PLP and loaded with the anti-inflammatory agent miR-21, were able to specifically deliver the nanospheres to inflammatory monocytes circulating in mice with myocardial IRI. These nanospheres enter the cytoplasm of monocytes via membrane fusion, facilitating the repair and reprogramming of the derived inflammatory macrophages. However, the treatment of myocardial IRI is further complicated by the challenges of myocardial enrichment and the low transfection efficiency in cardiomyocytes. To address this, the researchers developed a nanocomplex reversibly masked with a platelet-macrophage hybrid membrane [70]. This innovative nanocomplex can effectively deliver Sav1 siRNA (siSav1) to cardiomyocytes, inhibiting the Hippo signaling pathway and promoting cardiomyocyte regeneration. These novel nanoplatforms offer promising new solutions for the treatment of myocardial IRI by enhancing the efficiency and specificity of macrophage reprogramming. Further optimization of these nanoplatforms is expected to improve inflammation management, tissue repair, and patient outcomes in the future.

### Other cells

B cells, as key immune effector cells, primarily regulate the host’s immune response through the secretion of specific antibodies and antigen presentation [71]. During IRI, B cells not only participate in the damage cascade mediated by both innate and adaptive immunity, but also influence the recruitment and polarization of macrophages by secreting chemokines such as CCL2 and CXCL1. In turn, activated macrophages release pro-inflammatory cytokines, including IL-6 and TNF-α, which enhance the antigen-presenting capacity of B cells [72]. As the inflammatory response progresses, these cells migrate to distal organs, contributing to secondary immune-mediated tissue damage. However, endogenous regulatory mechanisms exist to counteract this immune-mediated tissue injury. Nakamoto et al. demonstrated that DCs upregulate IL-13 expression via the PGE2/EP3 signaling pathway [73], driving macrophage polarization from the M1 to the M2 phenotype, thereby promoting tissue repair. Furthermore, under hypoxic conditions, activation of the key transcription factor HIF-1α in macrophages triggers metabolic reprogramming, promoting the production and secretion of lactic acid. High concentrations of lactic acid can inhibit the maturation and activation of DCs, impairing their antigen-presenting function [74], and thus limiting the excessive expansion of the inflammatory response.

In addition to classic antigen-presenting cells, the immune function of eosinophils has gained increasing attention. Traditionally, eosinophils were thought to be primarily involved in parasitic infections and allergic reactions. However, recent studies have shown that within 24 h after IRI in the mouse liver, eosinophils are rapidly recruited to the liver, where they activate the IL-4Rα signaling pathway by secreting IL-4. This activation induces macrophages to secrete HB-EGF [75], which further promotes the regeneration and repair of damaged liver cells. Thus, various immune cells—such as B cells, dendritic cells, eosinophils, and macrophages—interact through complex signaling networks to both amplify and suppress the inflammatory response. Additionally, mast cells [76], γδ T cells [77], myeloid-derived suppressor cells [78], endothelial cells, and mesenchymal stem cells [79] play key roles in regulating the inflammatory response and maintaining the balance of the immune microenvironment. A deeper understanding of the interactions among these cells will be essential for fully elucidating the molecular mechanisms of IRI (Fig. 2).

## Macrophages and organ IRI

### Macrophages and cerebral IRI

**Fig. 3:**
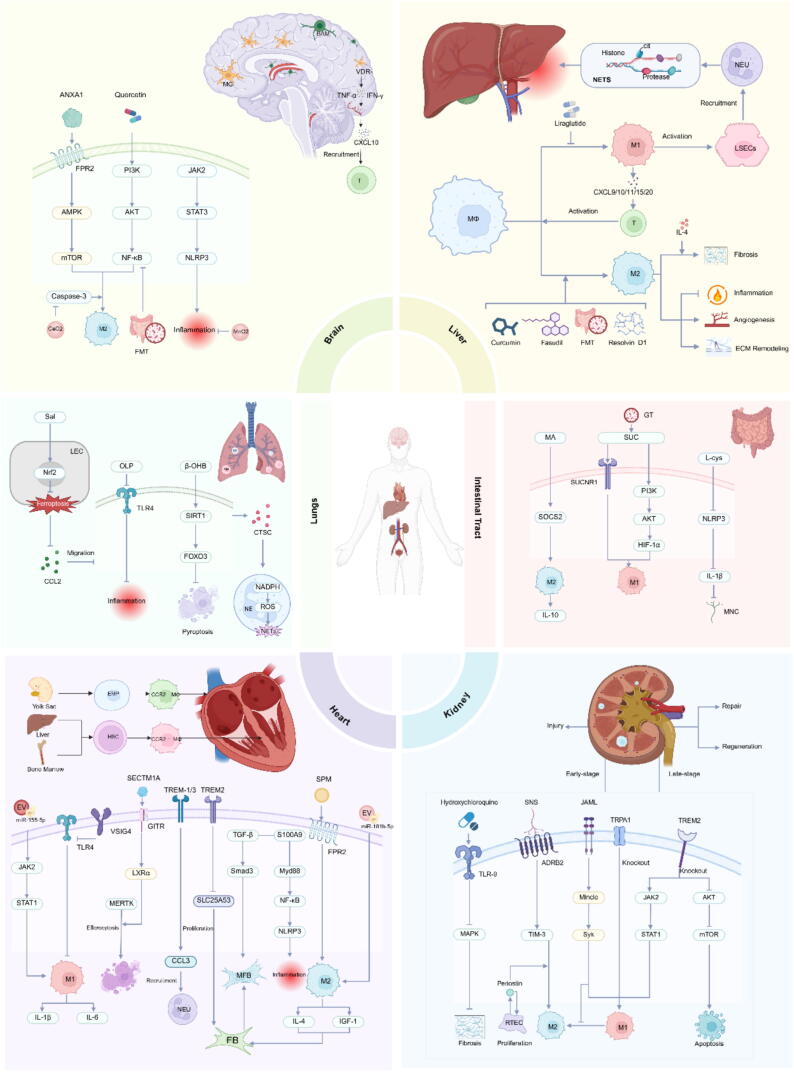
The role of macrophages in organ IRI. This illustration depicts the multifaceted roles of macrophages in IRI across various organs, including the brain, lung, heart, kidneys, liver and intestines. It highlights key signaling pathways and mechanisms involved in macrophage activation, polarization, and their contributions to inflammation, tissue repair, and regeneration in different organ systems.

IRI plays a critical role in stroke pathophysiology, with its hallmark feature being the secondary damage that occurs when blood flow is restored after ischemia. During both the ischemic and reperfusion stages, brain tissue undergoes a complex series of cellular, biochemical, and metabolic changes. These include excessive production of intracellular reactive ROS and reactive nitrogen species (RNS), calcium overload, glutamate neurotoxicity, inflammatory responses, and apoptosis [80].In this context, the activation of the immune system and inflammatory response is a key pathological aspect of cerebral IRI. Macrophages, as crucial immune cells, not only regulate the intensity and duration of the inflammatory response through the secretion of pro-inflammatory or anti-inflammatory factors, but also directly influence neuronal survival, the repair of the BBB, and the regeneration of brain tissue. This is achieved through their interactions with other cell types, including neurons, glial cells, and endothelial cells (Fig. 3) [81].

#### Source and recruitment of macrophages

Tissue-resident macrophages in the central nervous system (CNS) primarily consist of microglia and CNS-associated macrophages (CAMs). Microglia reside within the brain parenchyma, whereas CAMs are distributed at CNS interfaces, including the perivascular spaces, leptomeninges, and choroid plexus [82]. CAMs can be further classified based on anatomical location into dural macrophages (dmΜΦ), subdural or leptomeningeal macrophages (sdΜΦ), stromal choroid plexus macrophages (cpΜΦ), suprachoroidal plexus macrophages (cpepiΜΦ), and perivascular macrophages (PVMs) [83]. dmΜΦ exhibit a unique dual origin: an MHCII-positive subpopulation derived from bone marrow-derived monocytes, and an MHCII-negative subpopulation originating from embryonic progenitor cells, which are long-lived. PVMs and sdΜΦ surrounding the periaqueductal region and blood–brain barrier are also long-lived and maintained independently of circulating monocytes [84]. In contrast, cpΜΦ in the choroid plexus display shorter lifespans due to continuous replenishment from blood-derived monocytes [85], highlighting the complexity of origin, distribution, and renewal dynamics among brain-resident macrophages. Compared with CAMs, whose characteristics remain less understood, microglia have been more extensively studied. During development, the heterogeneity of microglia gradually decreases, resulting in reduced diversity in adulthood [86]. Microglia continuously monitor their surrounding microenvironment via surface receptors, which influence their behavior and function. Additional regulatory factors include sex, age, and signals derived from the periphery [87]. Moreover, intrinsic genetic programs also contribute to the regulation of microglial states and functions [88].

During cerebral IRI, disruption of the blood–brain barrier facilitates the infiltration of peripheral monocytes into brain tissue, where they differentiate into macrophages. These blood-derived monocytes/macrophages infiltrate the brain during the hyperacute phase of stroke (<24 h). Studies using chimeric mice with enhanced fluorescent protein-labeled bone marrow have demonstrated that blood-derived macrophages peak around day 7, followed by a gradual decline in their numbers [89]. In humans, histological analysis and positron emission tomography have revealed a large accumulation of activated microglia within 24–48 h post-stroke, persisting for several weeks and primarily localizing to the periphery of the ischemic core [90]. In the chronic phase, M2-polarized microglia promote neuroplasticity and neurogenesis through the secretion of various neurotrophic factors [91]. Immune cells exhibit distinct transcriptomic profiles at different stages of injury: microglia display a robust proliferative response by day 2, which diminishes by day 14. Meanwhile, blood monocytes expressing high levels of Ly6C and CCR2 gradually lose monocyte-specific markers and acquire gene expression profiles characteristic of tissue-resident macrophages [92]. These peripherally derived macrophages, together with resident microglia, contribute to the neuroinflammatory response by releasing effector molecules and recruiting additional immune cells. Emerging evidence indicates that these cells are highly plastic, capable of adopting diverse phenotypes and performing various functions in response to specific microenvironmental cues [93].

#### Macrophage polarization and function regulation in cerebral IRI

The polarization state and function of macrophages are intricately regulated by multiple signaling pathways and molecular networks. Classical pathways such as NF-κB, JAK/STAT, and MAPK play crucial roles in macrophage polarization. The gut microbiota is also a key regulator in maintaining macrophage development and function. Li et al. found that FMT can target and regulate the ERK and NF-κB signaling pathways, inhibiting microglial polarization toward the M1 phenotype [94]. On the other hand, quercetin induces macrophage polarization towards the M2 phenotype by modulating the PI3K/Akt/NF-κB signaling pathway, effectively alleviating cerebral IRI [95]. More recently, Zhu et al. found that NLRP3 inflammasomes are present in neurons and activated macrophages in the ischemic cortex. Janus kinase inhibitors reduce NLRP3 inflammasome activation by inhibiting the JAK2/STAT3 pathway, thereby improving ischemic stroke injury and neuroinflammation [96].

In addition to the classic signaling pathways, Annexin A1 (ANXA1), a calcium-dependent phospholipid-binding protein, plays a critical role in regulating inflammation and tissue repair. ANXA1 not only inhibits inflammatory responses but also promotes the clearance of cell debris. Studies have shown that ANXA1 and its mimetic peptide, Ac2-26, activate the AMPK-mTOR signaling pathway through interaction with the FPR2/ALX receptor, which promotes the conversion of macrophages from the pro-inflammatory M1 phenotype to the anti-inflammatory M2 phenotype. This conversion effectively mitigates tissue damage caused by cerebral IRI [97].Additionally, the role of vitamin D in cerebral IRI has garnered considerable attention. Population-based and cohort studies have shown that vitamin D deficiency is closely associated with the occurrence, progression, and prognosis of acute ischemic stroke [98]. Recent findings indicate that, following cerebral ischemia, the expression of the vitamin D receptor (VDR) in macrophages surrounding the infarct area is significantly upregulated. In the absence of VDR, macrophages release large amounts of inflammatory factors, such as TNF-α and IFN-γ, which stimulate endothelial cells to release CXCL10. This cascade results in the destruction of the BBB and enhances the infiltration of peripheral T lymphocytes, exacerbating brain damage and leading to more severe dysfunction [99].However, further research is needed to explore whether VDR signaling varies based on gender and age differences. In recent years, therapeutic strategies targeting macrophage polarization, such as small molecule drugs, signal pathway inhibitors, and biologically active molecules, have emerged as promising directions in stroke treatment. However, their mechanisms of action and clinical translation potential require further investigation and validation.

Reperfusion injury remains a significant challenge in improving the survival of neurons after ischemic injury, and existing treatments are often limited to targeting a single pathological process, lacking comprehensive neuroprotective effects. To address this, Li et al. developed a macrophage membrane-coated honeycomb MnO2 nanosphere [100]. This biomimetic nanoparticle is designed to specifically bind to cell adhesion molecules, which are highly expressed on the damaged vascular endothelium, through macrophage membrane proteins. As a result, these nanoparticles are selectively enriched in the damaged brain tissue, improving the pro-inflammatory microenvironment and promoting neuronal survival. The function and polarization state of macrophages are crucial factors determining the efficacy of these biomimetic nanoparticles. Additionally, with the rapid advancement of nanotechnology in the biomedical field, nanoenzymes have emerged as a novel class of materials. For example, CeO2 nanoenzymes have shown promise in reducing infarct size and improving cognitive function by decreasing Caspase 3 levels [101]. These nanoenzymes also induce the polarization of macrophages toward the M2 phenotype, which is associated with anti-inflammatory and tissue repair functions. As research continues to evolve, the performance of these nanoenzymes is expected to be further optimized, expanding their potential application across a broader range of diseases.

### Macrophages and lung IRI

Lung IRI is a common pathological process observed in lung transplantation, pulmonary embolism, and extracorporeal cardiac surgery. In lung transplantation, Lung IRI is a major cause of postoperative morbidity and mortality, primarily by disrupting the alveolar-capillary permeability barrier, promoting plasma exudation, causing interstitial edema and hemorrhage, and ultimately leading to ventilatory dysfunction [102]. Therefore, the development of effective strategies for preventing Lung IRI to improve both the short- and long-term prognosis of lung transplantation has become a key focus of current research.

#### Heterogeneity and function of macrophage subpopulations in the lungs

The development and differentiation of alveolar macrophages is a multistage process. These tissue-resident macrophages originate from distinct precursor cells [103], which mature and differentiate into tissue-resident alveolar macrophages (TR-AM) in the lung microenvironment after birth. Notably, lung macrophages exhibit significant heterogeneity. Under homeostatic conditions, alveolar macrophages in the lumen are defined as TR-AM, and their populations are primarily maintained through self-renewal and local proliferation. However, during IRI, monocytes are recruited into the inflammatory alveolar region, where they differentiate into recruited monocyte-derived TR-AM. In contrast, interstitial macrophages are located in the lung parenchyma, express CD64, CD11b, and CD11c, but lack Siglec-F expression [104]. Siglec-F has been shown to regulate immune cell activity and function by binding to its ligands, thereby influencing the onset and progression of the inflammatory response, a role distinct from that of TR-AM [105]. This difference in phenotype and distribution highlights the diversity and specialization of macrophage subpopulations in the lung. In lung IRI, macrophages finely regulate the inflammatory process, mediate tissue repair, and balance the immune response through dynamic polarization and precise phagocytosis. Five activated macrophage phenotypes have currently been identified, including M1-type, M2-type, CD169 + macrophages, TCR + macrophages, and tumor-associated macrophages [106]. In the acute phase of acute lung injury caused by various etiologies, M1 macrophages promote inflammation and exacerbate tissue damage, while M2 macrophages facilitate lung tissue repair by producing anti-inflammatory cytokines and promote fibrosis in later stages [107,108]. In the E. coli-induced Lung IRI mouse model, human mesenchymal stromal cells reduced bacterial burden in the lungs and improved animal survival, likely by enhancing macrophage phagocytosis [109]. Additionally, lung macrophages constitute the first line of defense against airborne particles and microorganisms, playing an irreplaceable role in maintaining immune homeostasis through antigen presentation, cytokine secretion, and tissue repair [110].

### Therapeutic Strategies for targeting macrophages

During pulmonary IRI, the body's response to inflammation and cellular injury is complex and involves the coordination and balance of multiple signaling pathways. Rhodiola rosea glycosides inhibit iron death in lung epithelial cells by activating the Nrf2 signaling pathway and decrease CCL2 expression to reduce macrophage chemotaxis [111], while β-OHB activates the SIRT1-FOXO3 signaling pathway to inhibit alveolar macrophage pyroptosis [112], and oleuropein reduces macrophage macrophage injury [113], all of these mechanisms can alleviate lung IRI. AMs also are involved in the regulation of neutrophil chemotaxis and infiltration, and it has recently been found that AMs significantly promote the formation of NETs through secretion of tissue protease C via the NADPH oxidase- and p38 MAPK-dependent reactive ROS-generating pathways which could be a novel therapeutic target for primary graft dysfunction [114]. All of these approaches are expected to be utilized in the future with the treatment of clinical lung IRI injury to improve lung function and survival.

### Macrophages and myocardial IRI

Myocardial IRI is a leading cause of mortality worldwide. It typically occurs during the stage of blood flow recovery following acute coronary artery occlusion, such as after myocardial infarction and reperfusion, as well as during various heart surgeries, organ transplants, and post-cardiac arrest resuscitation. Among these, acute reperfusion following myocardial infarction is the primary cause of myocardial IRI. Although current surgical and pharmacological treatments can improve patient symptoms and survival rates to some extent, many patients eventually develop adverse ventricular remodeling, which can progress to chronic heart failure. Macrophages are the most abundant immune cell population in cardiac tissue, and a deeper understanding of the complex balance between inflammation and fibrosis is crucial for reducing the occurrence of adverse events following myocardial IRI (Fig. 3), such as malignant arrhythmias, the no-reflow phenomenon, myocardial stunning, and the expansion of myocardial necrosis.

#### Classification and function of resident cardiac macrophages

Resident cardiac macrophages can be categorized into two subgroups: CCR2^-^ and CCR2^+^. Following tissue damage, CCR2^+^ macrophages secrete IL-1β, an inflammatory cytokine associated with cardiovascular disease. CCR2^-^ macrophages, primarily derived from the embryo, promote angiogenesis and cardiomyocyte proliferation. In pathological conditions such as acute myocardial infarction, most CCR2^-^ macrophages undergo apoptosis [115], while CCR2^+^ macrophages rapidly proliferate and infiltrate the lesion site. On one hand, these inflammatory macrophages limit secondary necrosis and prevent heart rupture by clearing damaged tissue. On the other hand, they upregulate the activity of key transcription factors, including NF-κB, STAT1, and HIF1α [116], forming a positive feedback loop that further amplifies inflammation. This sustained inflammatory response is thought to exacerbate the loss of myocardial collateral circulation and the expansion of the infarct, ultimately leading to cardiac dysfunction and adverse remodeling. Therefore, myocardial macrophages, as a multifunctional and dynamically changing population of immune cells, and their different subsets, play a complex and critical role in the development, maintenance of homeostasis, and stress response of the heart.

#### Macrophage involvement in myocardial IRI and repair post-injury

In the early phase of myocardial IRI, macrophages predominantly exhibit a proinflammatory phenotype, driving myocardial injury through various mechanisms, including the initiation of inflammatory responses, metabolic reprogramming, and cellular interactions. CCR2 + macrophages are the major proinflammatory subset during this phase, and Ly6Chigh monocytes are recruited to infarct zones via CCR2/CCL2 signaling. These monocytes further differentiate into CCR2 + MHC-IIhigh macrophages, replacing the resident macrophages that are lost. These recruited CCR2 + macrophages play a critical role in the inflammatory phase following myocardial infarction [115]. In contrast, in cardiac transplantation, tissue-resident CCR2 + macrophages promote CCL3 production via the TREM-1/3 signaling pathway, which is essential for the recruitment of neutrophils and CCR2 + monocytes after transplantation, thereby driving the post-transplant inflammatory response [117]. Using single-cell sequencing, Shen et al. identified a heterogeneous subpopulation of S100a9hi macrophages that infiltrate the heart during early reperfusion. These macrophages activate the MyD88/NFκB/NLRP3 signaling pathway, amplifying the inflammatory response [3]. As myocardial IRI activates the inflammatory cascade, macrophages also regulate the inflammatory response through metabolic reprogramming. Activation of HIF-1α in ischemic and hypoxic cardiomyocytes [118] promotes increased glycolysis, leading to lactic acid accumulation, which further stimulates the production of downstream inflammatory factors and promotes macrophage polarization toward the M1 phenotype [119], thus exacerbating myocardial injury. Furthermore, macrophage heterogeneity is likely influenced by the microenvironment. For instance, myocardial IRI induces the release of extracellular vesicles (EVs), which carry miR-155-5p and deliver it to macrophages, promoting a proinflammatory phenotype via activation of the JAK2/STAT1 pathway [120].

Macrophage phagocytosis of apoptotic cells and activation of the anti-inflammatory cascade initiate the reparative phase of infarct healing. This phase is characterized by immune cell-mediated anti-inflammatory signals, fibroblast proliferation, and the formation of granulation tissue [116]. On day 3 after myocardial IRI, reparative M2-type macrophages gradually dominate. A substantial number of apoptotic cardiomyocytes require macrophages to clear their cellular debris through multiple rounds of rapid phagocytosis, a process primarily regulated by specific receptors on the macrophage membrane [3]. SECTM1A, a secreted transmembrane protein, was found by Wang et al.[121] to enhance macrophage phagocytosis by activating the GITR/LXRα signaling pathway, thereby attenuating myocardial IRI. In the absence of SECTM1A, an increase in inflammatory macrophages is observed. Additionally, macrophages can utilize MerTK receptors to recognize and clear dead cardiomyocytes, preventing secondary necrosis and further inflammatory responses [122]. Beyond its own anti-inflammatory and repair functions, M2 macrophage-derived EVs effectively reduce the recruitment of CCR2 + macrophages to infarct sites while promoting macrophage conversion to the M2 phenotype and supporting neovascularization. The function of M2-EVs is attributed to their abundant miR-181b-5p content, which regulates macrophage glycolysis and attenuates mitochondrial ROS production [123]. Metabolic adaptation is a crucial feature and prerequisite for macrophage phenotypic transition. Metabolite-mediated epigenetic modifications regulate this transition; for example, lactic acid accumulated after reperfusion promotes the transcription of repair genes such as Lrg1, Vegf-a, and IL-10 via H3K18 lactation modification, thereby enhancing the dual anti-inflammatory and pro-angiogenic activities of monocyte-macrophages [124]. Macrophages achieve metabolic reprogramming through epigenetic remodeling to orchestrate their phenotypic shifts. Epigenetic inhibition of TSC1, mediated by the interaction of the epigenetic factor NPM1 and the histone demethylase KDM5b, enhances mTOR-associated inflammatory glycolysis, maintaining the inflammatory phenotype of cardiac macrophages. Conversely, NPM1 deficiency shifts macrophage metabolism from an inflammatory glycolytic system to oxygen-driven mitochondrial energy production, thereby enhancing the repair function of cardiac macrophages [125].

In the late phase of myocardial IRI), M2-type macrophages further promote the polarization of peripheral macrophages toward a reparative phenotype by secreting factors such as IL-4, IGF-1, and TGF-β. Macrophage subpopulations involved in repair are classified into M2a, M2b, and M2c subtypes. Previous studies reported that the M2b subpopulation was not associated with tissue remodeling. However, a subsequent study found that reduced kinase activation of the platelet-derived growth factor receptor in mice injected with M2b macrophages prevented myocardial tissue remodeling after myocardial IRI [126]. Furthermore, recent studies have revealed the role of heterogeneous macrophage subpopulations in myocardial repair following IRI. A pro-fibrotic macrophage population marked by the expression of Spp1, Fn1, and Arg1 (referred to as Spp1 macrophages) showed increased expression after myocardial injury. Their differentiation was regulated by the platelet-derived chemokine CXCL4, and the deletion of CXCL4 suppressed Spp1 macrophage differentiation, attenuating fibrosis after cardiac injury [127]. Gong et al. found that the cytosolic function of TREM2 + macrophages infiltrating the infarcted and marginal zones was enhanced. TREM2 influenced the mitochondrial uptake of NAD + by inhibiting the expression of SLC25A53, which interrupted the TCA cycle and increased the production of itaconate. Itaconate, in turn, played a role in inhibiting cardiomyocyte apoptosis and promoting fibroblast proliferation, highlighting the important role of immunometabolism in post-infarction repair. Additionally, the S100a9hi macrophage subpopulation, which is involved in the inflammatory progression during the early stages of IRI, also induces fibroblasts to transdifferentiate into myofibroblasts through the TGF-β/p-Smad3 signaling pathway as the inflammatory response subsides, thereby accelerating myocardial fibrosis [3].

#### Therapeutic strategies targeting macrophages

Macrophage infiltration and polarization are particularly critical in heart failure and myocardial fibrosis following IRI. V-set and immunoglobulin domain-containing 4 (VSIG4) is an immune regulatory protein primarily expressed on macrophages and other antigen-presenting cells. Wang et al. [128] found that VSIG4 inhibits M1 polarization of macrophages by blocking activation of the TLR4 pathway, thereby preventing cardiomyocyte apoptosis. More recently, Li et al. [123] evaluated the plasticity and reparative effects of M2 macrophage-derived small extracellular vesicles (M2EV) in rat and porcine myocardial IRI models. They observed that M2EV prevented the imbalance in the CCR2^+^/CCR2^-^ macrophage ratio and inhibited the recruitment of CCR2^+^ macrophages to the myocardium after IRI, thereby promoting cardiac repair by enhancing revascularization. Although most interventions aim to suppress the inflammatory response after IRI, in some patients with impaired anti-inflammatory capacity, exacerbation of IRI may also occur. In line with this approach, preclinical studies have explored the therapeutic potential of improving cardiac function by promoting the reparative properties of immune cells. For example, specialized pro-resolving mediators (SPM) signal through formyl peptide receptor 2 (FPR2) to induce macrophages and neutrophils to polarize toward a resolving phenotype, thereby promoting wound healing [129]. Treatment of rats with the FPR2 agonist BMS-986235 can increase macrophage phagocytosis and clear neutrophils, thus preserving cardiac tissue and improving patient prognosis [130]. Future therapeutic strategies may improve the immune environment by modulating multiple immune mechanisms. Additionally, strategies targeting the interactions between different cell types and macrophages may offer a key approach for the prevention and treatment of myocardial IRI [54,55].

### Macrophages and hepatic IRI

Hepatic IRI commonly occurs in conditions such as hemorrhagic shock, trauma, hepatectomy, and liver transplantation. It not only directly causes liver dysfunction but also induces systemic damage by impairing the function of distal organs, thereby increasing postoperative mortality. Among parenchymal organs, the liver contains the highest proportion of macrophages. These liver macrophages can polarize into either M1 or M2 phenotypes in response to various cytokines, including IFNs, TLR ligands, and IL-4/IL-13. Both phenotypes are actively involved in regulating sterile inflammation in the liver and play critical roles in initiating, sustaining, and resolving hepatic IRI (Fig. 3) [131].

#### Macrophage polarization and hepatic IRI

During the early stage of reperfusion, hypoxia leads to accelerated hepatic ATP catabolism, resulting in the production of large amounts of reactive ROS and the release of DAMPs. In response, macrophages rapidly activate into the M1 phenotype and secrete high levels of pro-inflammatory cytokines such as TNF-α, IL-1β, and IL-6, which activate sinusoidal endothelial cells. This upregulates the expression of intercellular adhesion molecule 1 and vascular cell adhesion molecule 1, promoting the adhesion of neutrophils to endothelial cells and triggering an inflammatory cascade. Simultaneously, the recruited neutrophils activate platelets and form microthrombi by releasing NETs rich in DNA, histones, and proteases [132], which further exacerbate liver ischemia–reperfusion injury. In this process, M1 macrophages also recruit Th1 cells by secreting chemokines such as CXCL9, CXCL10, CXCL11, CXCL15, and CXCL20, inducing a Th1 immune response that further activates macrophages and enhances their phagocytic capacity, creating a positive feedback amplification loop. This interplay between innate and adaptive immunity constitutes a key mechanism of early pathological damage in liver IRI.

In contrast, M2 macrophages primarily exert a protective effect by mediating the secretion of anti-inflammatory factors, promoting angiogenesis, and participating in tissue repair and extracellular matrix reconstruction. Recent studies have shown that the absence of monocytes/macrophages inhibits angiogenesis in some mice following partial hepatectomy and delays liver regeneration [133]. However, when M2 macrophages are overactivated, they continue to secrete pro-fibrotic factors such as TGF-β and PDGF, exacerbating liver damage. In this process, IL-4 promotes the conversion of arginine into polyamines and ornithine by activating the expression of arginase in M2 macrophages, leading to excessive extracellular matrix deposition. Studies have demonstrated that blocking IL-4 receptor signaling or using IL-4 receptor antibodies can reduce the degree of fibrosis in diseased tissues [134]. Additionally, the JAK2/STAT3 signaling pathway is a key molecular mechanism regulating the polarization of M2 macrophages. A study using a mouse model confirmed that Peroxisome proliferator-activated receptor-γ coactivator-1α can significantly improve IRI-induced liver fibrosis by inhibiting M2 macrophage polarization mediated by the IL-6/JAK2/STAT3 signaling pathway [135].

#### Therapeutic strategies targeting macrophages

IRI is a major clinical predictor of poor prognosis in patients undergoing partial hepatectomy and liver transplantation. A variety of drugs have been shown to promote the polarization of liver macrophages to the M2 phenotype, with curcumin, fasudil, and resolvin D1 demonstrating promising therapeutic effects [136]. Additionally, liraglutide has been found to inhibit the polarization of macrophages toward the inflammatory phenotype by activating the GLP-1 receptor [137], thus alleviating liver IRI. In addition to pharmacological interventions, the gut microbiota and its metabolites also play a pivotal role in regulating macrophage metabolic reprogramming. Lu et al. showed that antibiotic pretreatment significantly reduced the degree of IRI-induced liver injury, and this effect could be transferred to germ-free mice via fecal microbiota transplantation (FMT), suggesting a protective effect from intestinal microbiota depletion [138]. Further studies have revealed that elevated glutamine levels in the intestine lead to increased blood levels of α-ketoglutarate, which promotes M2 polarization of macrophages by enhancing the tricarboxylic acid cycle, increasing the number of M2 macrophages, and thus aiding in liver IRI repair. However, the application of oligomycin A inhibits the polarization of M2 macrophages, thereby reversing this protective effect. Therefore, potential therapies targeting macrophage metabolism, such as antibiotic treatments and novel immunometabolism regulators, could provide new avenues for treating liver IRI. These approaches are expected to improve liver function and enhance patient survival by modulating the gut microbiota and its metabolites.

### Macrophages and renal IRI

Renal IRI is a significant health concern that has garnered increasing global attention. However, effective prevention and treatment options remain limited. Macrophages, known for their phagocytic capabilities, exhibit considerable diversity and play essential roles throughout the different stages of renal IRI (Fig. 3). In the early stages, activated intrarenal macrophages contribute to tubular injury; as the injury progresses, these macrophages shift to primarily facilitate the repair and regeneration of renal tubular epithelium [139]. This dynamic process of macrophage polarization underscores their plasticity and highlights the regulatory influence of microenvironmental signals on the activation of tissue macrophages at both the organ and cellular levels.

#### Activation of pro-inflammatory macrophages during Initial kidney injury

During the early stages of renal injury, multiple molecular mechanisms regulate the pro-inflammatory activation of macrophages. Triggering receptor expressed on myeloid cells 2 (TREM2), an immune receptor found on the surface of myeloid cells, plays a critical role in this process. TREM2 deficiency has been shown to induce macrophage apoptosis by downregulating the Akt/mTOR signaling pathway. However, it also activates the JAK-STAT pathway, promoting macrophage polarization towards the M1 phenotype [140], which exacerbates acute tubular injury in mice with renal IRI. Beyond membrane receptors, ion channels are also essential in regulating macrophage function. Transient receptor potential ankyrin 1 (TRPA1), a non-selective calcium-permeable cation channel, is pivotal in detecting harmful environmental and internal stimuli, such as oxidative stress. Knockout of the TRPA1 gene increases the production of pro-inflammatory cytokines, such as IL-1β and TNF-α, by M1 macrophages [141], further aggravating renal inflammation and dysfunction following IRI. In recent years, junctional adhesion molecules (JAMs) have gained attention for their role in immune cell activation and inflammatory responses. Junctional adhesion molecule-like protein (JAML), a newly identified member of the JAM family, has been shown to maintain the M1 phenotype of macrophages via the Mincle/Syk signaling pathway. Huang et al. [142] demonstrated that in a mouse model of AKI induced by renal IRI, JAML not only sustains the M1 phenotype but also significantly inhibits the conversion of M1 to M2 macrophages, thereby exacerbating the necroinflammatory cycle.

#### Macrophage activation during kidney repair

In the later stages of kidney IRI, pro-inflammatory macrophages gradually transition to an anti-inflammatory phenotype. The sympathetic nervous system plays a crucial regulatory role in this process. Studies have shown that activation of the β2-adrenergic receptor (Adrb2) in macrophages effectively inhibits the LPS-induced systemic inflammatory response and mitigates renal IRI [143]. Moreover, Adrb2 signaling induces the expression of the T cell-associated molecule Tim3, which promotes macrophage polarization towards an anti-inflammatory phenotype. Additionally, macrophages contribute to tissue repair and remodeling by secreting growth factors, a process that is precisely regulated by multiple genes. Among these, transcription factor 3 (ATF3) is a key regulatory molecule. Its expression is linked to increased inflammatory responses and cytokine secretion, such as IL-6 [144,145]. There is a significant negative correlation between ATF3 and macrophage infiltration, suggesting that ATF3 may play a role in the pathophysiology of renal IRI by modulating macrophage recruitment. Furthermore, thyroid receptor interacting protein 13 (TRIP13), a critical renal stressor, influences the overall immune response by regulating macrophage infiltration, thereby promoting the repair of renal tubular epithelial cells [146,147].These findings provide an important theoretical foundation for the development of therapeutic strategies targeting macrophages.

#### Therapeutic strategies targeting macrophages

Successful regeneration of damaged epithelial cells and the transformation of macrophages into a reparative phenotype are believed to play a critical role in kidney recovery. Studies have shown that periostin, a protein derived from epithelial cells, exhibits a dual role in renal IRI. Early on, it inhibits epithelial cell death, preventing early tubular injury and worsening renal function [148]. In the later stages, periostin promotes the transformation of macrophages into the M2 phenotype, facilitating renal tubular repair. However, the persistent presence of pro-inflammatory macrophages can contribute to late-stage tubular atrophy and interstitial fibrosis. Zheng et al. demonstrated that hydroxychloroquine can inhibit macrophage activation and MAPK signaling through the TLR-9 pathway [149], ultimately reducing renal fibrosis following IRI. Moreover, CD206 + M2 macrophages are closely linked to renal fibrosis, with TGF-β1 playing a pivotal role in epithelial-mesenchymal transformation, extracellular matrix production, and tissue remodeling. Recent studies have confirmed that the TGF-β1 peptide-based inhibitor P144 blocks the polarization of macrophages into an M2-like phenotype and inhibits TGF-β1-induced macrophage migration in vitro [150], significantly improving renal fibrosis.

In addition to the studies mentioned above, existing data from mouse kidney IRI models indicate that the source, activation dynamics, and specific injury types of macrophages can directly influence the therapeutic outcome [151]. However, sequential renal biopsies are rarely performed on patients with ischemic renal injury in clinical practice, making it challenging to directly translate findings from mouse AKI models to human patients. In the future, researchers may establish a biomarker library for patients with ischemic renal injury through multicenter clinical studies, enabling cross-validation with mouse models. Additionally, by examining the specific functions of macrophages in different injury types and assessing their dynamic changes at various time points, the optimal treatment window can be identified, providing patients with more effective treatment options.

### Other organs

IRI is a complex pathological process that affects not only the brain, lungs, heart, liver, and kidneys but also vital organs such as the gut and pancreas. Intestinal IRI can disrupt intestinal barrier function, promote microbial translocation, and trigger systemic inflammatory responses, while pancreatic IRI can induce acute pancreatitis, tissue damage, and an inflammatory cascade. Macrophages in these organs play a crucial role in regulating tissue injury and repair by releasing inflammatory mediators and modulating the immune response during IRI.

Intestinal IRI is a severe pathology commonly observed in conditions such as strangulated bowel obstruction, trauma, and acute mesenteric ischemia. This injury leads to extensive necrosis of the mucosal epithelium, loss of villi and crypt structures, and consequently, testinal barrier dysfunction, with a mortality rate ranging from 50 % to 90 % [152]. Transplantation of intestinal organoids offers a promising therapeutic approach for treating mucosal injuries. Zhang et al. demonstrated that organoids, by secreting the metabolic substrate MA, promote M2 macrophage polarization and restore IL-10 levels in a SOCS2-dependent manner during intestinal IRI. This represents a novel metabolic-endocrine mechanism by which transplanted organoids regulate the immune microenvironment in the recipient, offering a potential treatment for intestinal IRI [153]. In addition, metabolic disorders of the gut microbiota are involved in the macrophage polarization process, as demonstrated by a recent study by Wang et al. In this study, after intestinal IRI in mice, gut microbiota-derived succinic acid promoted alveolar macrophage polarization toward the M1 phenotype during intestinal I/R through the SUCNR1 and PI3K/AKT/HIF-1α pathways. This polarization subsequently led to alveolar epithelial cell apoptosis and acute lung injury [154]. In another study, it was noted that L-cysteine attenuated intestinal IRI-induced neuronal damage in the interosseous plexus, with this protective effect largely dependent on the NLRP3-IL-1β inflammasome pathway in macrophages [155].

The current understanding of pancreatic IRI remains limited, primarily due to the complexity of its anatomical and physiological structure. Existing experimental models and technical tools often fail to effectively simulate the actual clinical scenario. While animal experiments can provide valuable data, the validity of their translation to humans is frequently questionable. However, it has been shown that Oleuropein can inhibit inflammation and oxidative stress by modulating the HMGB1/NF-κB signaling pathway, thereby alleviating pancreatic IRI [156]. Clearly, many questions remain unanswered regarding the mechanisms of macrophage involvement in pancreatic IRI, which offers a clear direction for future research.

## Clinical applications of macrophage-targeted therapies

The drugs listed in the Table 1 highlight the therapeutic potential of targeting macrophages in the treatment of clinical IRI. These agents modulate macrophage activity through various mechanisms, including the regulation of inflammation and modulation of key signaling pathways. Autologous transplantation of M2-polarized macrophages exerts anti-inflammatory effects primarily through the secretion of cytokines such as interleukin-10 (IL-10) and transforming growth factor-beta (TGF-β) [157]. Coenzyme Q10, a naturally occurring ubiquinone in humans and animals, has been shown to reduce neuroinflammation following IRI by inhibiting macrophage recruitment and suppressing the NLRP3/IL-1β signaling pathway [158]. Additionally, it exerts an inhibitory effect on myocardial ischemia–reperfusion injury. Colchicine, a well-known alkaloid, when delivered via nanoparticles, mitigates inflammatory cascades by modulating macrophage polarization from M1 to M2 phenotype and inhibiting the TLR4/NF-κB/NLRP3 signaling axis [159]. These clinical interventions demonstrate the effectiveness of both cell-based therapies and pharmacological approaches in modulating macrophage responses during IRI.

**Table 1:** Clinical trials targeting macrophages in organ ischemia–reperfusion injury.

Therapy	Type	Drug	Indications	Organ	Phase	NCT number	Reference
Direct inhibitors	Cellular Therapy Product	Autologous M2 macrophages	Stroke	Brain	Completed	NCT01845350	[157]
	Antioxidant	Coenzyme Q10	Myocardial infarction	Myocardium	−	ChiCTR2100045256	[158]
			An elective vascular operation	Myocardium	I	NCT03956017	[163]
			Stroke	Brain	III	IRCT20210907052400N1	[164]
	Alkaloid	Colchicine	Myocardial infarction	Myocardium	VI	NCT05709509	NA
		Theophylline	Acute Ischemic Stroke	Brain	Completed	2013–001989-42	[165]
		Berberine	Post- PCI	Myocardium	−	ChiCTR1900023731	NA
	Small molecules	Tacrolimus	Hepatic transplant	Hepatic	Completed	NCT01887171	NA
		Sirolimus	Kidney transplant	Kidney	Completed	NCT00866879	[166]
Indirect inhibitors	Metabolic modulator	Heme arginate	Kidney transplant	Kidney	Completed	NCT01430156	[160]
	Small molecules	Deoxyspergualin	Kidney transplant	Kidney	Terminated	NCT01052259	NA
		Maraviroc	Stroke	Brain	II	NCT04966429	NA
	CD20	Rituximab	Kidney transplant	Kidney	Terminated	NCT01318915	NA

In addition to their direct effects, some drugs exert anti-inflammatory actions by modulating macrophage metabolism and regulating immune cell infiltration. Heme oxygenase-1 (HO-1) demonstrates antioxidant and anti-inflammatory properties through the degradation of heme, producing biliverdin and bilirubin. Macrophages, as the primary producers of HO-1, mitigate ischemia–reperfusion-induced oxidative stress injury by upregulating HO-1 expression. Pretreatment with hematoxylin arginine (HA) significantly enhances HO-1 expression in renal interstitial macrophages via activation of the Nrf2/ARE signaling pathway, thereby improving outcomes in Ischemia–Reperfusion Injury (IRI) [160]. Furthermore, maraviroc, a CCR5 inhibitor, has been shown to improve neurological function following stroke by reducing the infiltration of peripheral monocytes [161]. Rituximab, an anti-CD20 monoclonal antibody, has been shown to modulate the immune response in kidney transplantation by inducing cytokine secretion, particularly macrophage inflammatory protein (MIP)-1β, which in turn affects macrophage activity [162].

Although significant progress has been made in targeting macrophages for the treatment of Ischemia–Reperfusion Injury (IRI), future studies should further investigate the dynamic changes of macrophage subpopulations during IRI and develop spatiotemporally specific therapeutic strategies. Additionally, the use of nanocarriers to enhance drug penetration across the blood–brain barrier may improve the precision and efficacy of treatment regimens.

## Summary

Macrophages play a critical and multifaceted role in the pathophysiology of IRI. They contribute to disease progression through classical mechanisms such as phagocytosis and the secretion of pro-inflammatory cytokines. Additionally, macrophages influence disease outcomes by mediating processes like the formation of METs. Throughout the various stages of IRI, macrophages exhibit considerable plasticity and functional diversity, which can either exacerbate tissue damage or promote repair. Recent advancements in understanding macrophage functions and their regulatory mechanisms have uncovered new therapeutic strategies for IRI. For instance, approaches aimed at modulating macrophage polarization and inhibiting excessive METs formation have demonstrated promising therapeutic potential in preclinical models.

However, many aspects of macrophage involvement in IRI remain poorly understood. Specifically, the interactions between macrophages and other immune cells, the precise roles of different macrophage subpopulations in the disease process, and the development of precision therapies require further investigation. Future research should focus on addressing these critical questions and advancing more effective treatment strategies, ultimately improving patient outcomes. Additionally, translating basic research findings into clinical applications is essential, and further clinical trials are needed to assess the safety and efficacy of these emerging therapies.

## Compliance with Ethics Requirement

This review does not involve any studies with human or animal subjects.

## Declaration of competing interest

The authors declare that they have no known competing financial interests or personal relationships that could have appeared to influence the work reported in this paper.
